# The mechanism and cause of anterior cruciate ligament tear in the Korean military environment

**DOI:** 10.1186/s43019-019-0015-1

**Published:** 2019-11-28

**Authors:** Joosuk Ahn, Byungseop Choi, Yong Seuk Lee, Ki Woung Lee, Jung Woo Lee, Beom Koo Lee

**Affiliations:** 1 Department of Orthopaedic surgery, Armed Forces Capital Hospital, 81, Saemaeul-ro 177beon-gil, Bundang-gu, Seongnam-si, Gyeonggi-do Republic of Korea; 2 Department of Orthopaedic Surgery, Seoul National University Bundang Hospital, Seoul National University College of Medicine, 82, Gumi-ro 173beon-gil, Bundang-gu, Seongnam-si, Gyeonggi-do Republic of Korea; 3 Department of Orthopaedic Surgery, Hallym Sacred Heart Hospital, Hallym University College of Medicine, 22, Gwanpyeong-ro 170beon-gil, Dongan-gu, Anyang-si, Gyeonggi-do Republic of Korea; 4 Department of Orthopaedic Surgery, Gil Hospital, Gachon University of Medicine and Science, 21, Namdong-daero 774beon-gil, Namdong-gu, Incheon, Republic of Korea

**Keywords:** Anterior cruciate ligament, Injury mechanism, Injury cause, Military environment, Injury prevention program

## Abstract

**Purpose:**

Anterior cruciate ligament (ACL) injury is very common but few studies have analyzed the injury mechanism and cause of ACL tear in a specific environment such as a military institution. The purpose of this study was to analyze the injury mechanism and cause of ACL injury in the military environment. Additionally, this study could provide outcomes that may aid future studies on prevention of ACL injury in military personnel.

**Materials and methods:**

This study retrospectively analyzed 168 patients who sustained ACL tear while in military service and underwent ACL reconstruction surgery in a military hospital. Analysis of the injury mechanism and the cause was evaluated by analyzing the medical records. Knee magnetic resonance imaging analysis was also conducted for further evaluation of associated injury.

**Results:**

The majority of ACL injuries in the military environment occurred through non-contact injury. Changing direction (46.4%) was the most common lower-leg position, followed by landing with the knee in a valgus position (26.8%). The activity undertaken at the time of injury was exercise in 76.2% of cases and military training/daily activities in 23.8% of cases. The incidence of ACL injury was higher in the soldier compared to the officer group during exercise (*P* = 0.017). Soccer was the most common activity at the time of injury (54.1%), followed by military training/daily activities, futsal, and basketball. The most common injury time was between 30 and 60 min after the start of exercise. Commonly associated injury sites were the medial meniscus and the medial collateral ligament.

**Conclusions:**

The main mechanism of ACL injury occurring in the military environment was non-contact injury, especially on changing the direction of the lower leg. Soccer was the most frequent activity at the time of the injury. These findings suggested that preventive strategies against ACL injury in the military environment could effectively reduce the incidence of ACL injury.

## Introduction

Anterior cruciate ligament (ACL) injury is common in the general population, with a reported incidence of 100,000–250,000 per year in the USA [1–5]. It is also common in the military environment and there are a reported 2.1 ACL injuries per 1000 people in the Finnish army every year [6]. This not only produces a large number of patients, but also results in loss of military manpower and combat capabilities. Therefore, it is strategically important not only to provide effective treatments, but also to prevent injury.

For the prevention of ACL injury it is crucial to identify the commonly associated injury mechanism and then apply strategies to lower the risk. Several previous studies in the sports environment have not only analyzed the risk factors and injury mechanisms associated with ACL injuries through biomechanics studies and sports video analysis [7], but have also reported that the application of prevention programs developed through the analysis was effective in preventing ACL injuries [4, 8–11].

However, there is distinct lack of literature that has focused specifically on ACL injury in military personnel. The military environment is characterized by excessive exposure to strenuous physical activity, heavy lifting, and repeated stress activities. These factors create an environment markedly different from that of the general population. Therefore, the purpose of this study was to analyze the injury mechanism and cause of ACL injury in the Korean military environment.

## Materials and methods

Patients who received ACL reconstruction surgery between August 2015 and January 2017 at a single institution (military hospital) were retrospectively reviewed, and 233 patients who underwent ACL reconstruction for ACL tear were identified. Of these, 65 patients were excluded as follows: (1) patients who underwent any previous surgical procedures on the affected side of the knee before ACL reconstruction (*n* = 51); (2) patients who were diagnosed with chronic tear of the ACL on magnetic resonance imaging (MRI), and thus were unable to confirm the time of the injury (*n* = 1); and (3) patients with insufficient medical records (*n* = 13). A total of 168 patients were included for final analysis. This study was approved by the institutional review board of our institution.

Data collected consisted of the following: (1) demographic factors - age, sex, affected side of the knee, height, weight, and military rank; (2) ACL injury mechanism at the time of injury; (3) associated injuries of cartilage and ligaments as seen on MRI; and (4) factors associated with activity at the time of the injury, including type of activity and time from the start of activity to injury.

For documentation of the injury mechanism, injury type was classified into contact and non-contact injury, according to the presence of a direct impact onto the lower extremities [7, 12–14]. In addition, according to the position of the lower leg at the time of injury, contact injury was classified as valgus, varus, or hyperextension, and non-contact injury was classified as landing in valgus, varus, or changing direction [7, 12–14]. The duration of exercise was divided into 30-min intervals in order to investigate the relationship between the duration of exercise and the incidence of ACL injury. Associated injuries such as meniscus tears and other ligamentous injuries were evaluated through MRI.

For statistical analysis, patients were initially divided into the officer group (*n* = 90, 53.6%) and the soldier group (*n* = 78, 46.4%). The mechanisms of ACL injury, factors associated with the injury, and activity at the time of injury were analyzed by comparing the two groups. We tested to confirm normal distribution of the data and equality of variances. Data were statistically analyzed using the *t* test, Mann-Whitney test, Fisher’s exact test, or the chi-square test, as appropriate. The Bonferroni correction for multiple comparisons was applied to confirm if there were significant differences between the subgroups. Data were analyzed using SPSS 25.0 software (SPSS Inc., Chicago, IL, USA), and with *P* < 0.05 as the significance level.

## Results

There were 168 patients with sufficient data available for detailed analysis. A summary of the patient demographics can be seen in Table 1. Between the officer group and the soldier group, there was no statistically significant difference in patient characteristics except for age, weight and body mass index (BMI). The mean age of the officer group was around 10 years older than that of the soldier group (*P* < 0.001). Also, mean weight and BMI were statistically significantly higher in the officer group (*P* = 0.045 and *P* = 0.002, respectively).

**Table 1:** Patients’ characteristics

	Total (*n* = 168)	Officer group (*n* = 90)	Soldier group (*n* = 78)	*P* value
Mean age, years	27.1	31.6	21.9	< 0.001
Sex^a^				–
Male	167 (99.4%)	89 (98.9%)	78 (100%)	
Female	1 (0.6%)	1 (1.1%)	–	
Affected side				0.138
Right	90 (53.6%)	53 (58.9%)	37 (47.4%)	
Left	78 (46.4%)	37 (41.1%)	41 (52.6%)	
Height	175.5 cm	174.9 cm	176.2 cm	0.078
Weight	76.2 kg	77.6 kg	74.6 kg	0.045
BMI	24.7 kg/m^2^	25.4 kg/m^2^	24.0 kg/m^2^	0.002
Injury pattern				0.976
Non-contact	142 (84.5%)	76 (84.4%)	66 (84.6%)	
Contact	26 (15.5%)	14 (15.6%)	12 (15.4%)	

*BMI* body mass index

^a^Statistical analysis of group difference in sex was not conducted, since the soldier group in Korea consisted only of men

The results showed that the overwhelming majority of ACL injuries occurred through non-contact injury, accounting for 84.5% of the total. Of the 84.5%, the most common lower-leg position at the time of injury was changing direction (46.4%), followed by landing in the valgus position (26.8%) (Table 2).

**Table 2:** Analysis of the anterior cruciate ligament injury mechanism

	Total (*n* = 168)	Officer group (*n* = 90)	Soldier group (*n* = 78)	*P* value
Non-contact	142 (84.5%)	76 (84.4%)	66 (84.6%)	0.938
Changing direction	78 (46.4%)	42 (46.7%)	36 (46.2%)	
Landing in valgus	45 (26.8%)	25 (27.7%)	20 (25.6%)	
Landing in varus	19 (11.3%)	9 (10.0%)	10 (12.8%)	
Contact	26 (15.5%)	14 (15.6%)	12 (15.4%)	
Valgus	9 (5.4%)	6 (6.7%)	3 (3.8%)	
Varus	15 (8.9%)	7 (7.8%)	8 (10.3%)	
Hyperextension	2 (1.2%)	1 (1.1%)	1 (1.3%)	

Analysis of activities at the time of injury showed that 76.2% were during exercise and 23.8% were during military training/daily activities. Furthermore, when compared with the officer group, the incidence of ACL injury was higher in the soldier group during exercise (*P* = 0.017; Table 3). Soccer was the most common activity associated with ACL injury, at 54.1%, followed by military training/daily activities, futsal, and basketball.

**Table 3:** Analysis of an association between anterior cruciate ligament injury and the activity at the time of injury

	Total (*n* = 168)	Officer group (*n* = 90)	Soldier group (*n* = 78)	*P* value
Exercise	128 (76.2%)	62 (68.9%)	66 (84.6%)	0.017
Military training/daily activities	40 (23.8%)	28 (31.1%)	12 (15.4%)	

There was no significant difference between the incidence of non-contact injury and contact injury in sports/exercise and military training/daily activities. However, an association between lower leg position at the time of injury and the activity during injury was statistically significant (*P =* 0.001; Table 4). In particular, the highest incidence of ACL injury occurred when changing direction during exercise.

**Table 4:** Analysis of an association between the anterior cruciate ligament injury mechanism and the activity

	Total (*n* = 168)	Exercise (*n* = 128)	Military training/ daily activities (*n* = 40)	*P* value
Non-contact	142 (84.5%)	108 (84.4%)	34 (85.0%)	0.001
Changing direction	78 (46.4%)	67 (52.3%)	11 (27.5%)	
Landing in valgus	45 (26.8%)	34 (26.6%)	11 (27.5%)	
Landing in varus	19 (11.3%)	7 (5.5%)	12 (30.0%)	
Contact	26 (15.5%)	20 (15.6%)	6 (15.0%)	
Valgus	9 (5.4%)	7 (5.4%)	2 (5.0%)	
Varus	15 (8.9%)	11 (8.6%)	4 (10.0%)	
Hyperextension	2 (1.2%)	2 (1.6%)	0	

The most frequent injury time point was between 30 and 60 min after the start of exercise (46.1%), followed by 0–30 min (39.8%). Subgroup analysis showed that the officer group more often tended to be injured within 30 min, and the soldier group tended more often to be more injured between 30 and 60 min after the start of injury; however, this was not statistically significant (Table 5).

**Table 5:** Analysis of an association between anterior cruciate ligament injury and the duration of exercise

Duration of exercise	Total (*n* = 128)	Officer group (*n* = 62)	Soldier group (*n* = 66)	*P* value
<30 min	51 (39.8%)	30 (48.4%)	21 (31.8%)	0.172
30 ~ 60 min	59 (46.1%)	27 (43.5%)	32 (48.5%)	
>60 min	18 (14.1%)	5 (8.1%)	13 (19.7%)	

Analysis of associated injuries was conducted by MRI, and was divided into meniscus and other ligamentous injuries. Excluding isolated ACL injuries, the most frequently associated injuries were to the medial meniscus (22.6%), followed by the lateral meniscus (20.5%) and both menisci (10.4%). The most frequent ligamentous injuries were to the medial collateral ligament (10.4%), followed by the medial and lateral collateral ligaments (9.8%) and the lateral collateral ligament (6.1%).

## Discussion

There were several noteworthy findings in this study. First of all, the main mechanism of ACL injury that occurred during military service was due to non-contact injury, most commonly by changing the direction of the lower leg at the time of injury, followed by landing with the knee in the valgus position. These results are similar to those reported in the sporting field [2, 8, 10, 12–17]. Moreover, the greatest number of ACL injuries occurred during exercise while changing direction, especially during soccer. Although the most frequent time of injury was between 30 and 60 min after the start of exercise, this was not statistically significant.

It is well-established that ACL injury commonly occurs in young athletes [13, 14, 18]. It was previously reported that the incidence of ACL injury in the military population is about 10 times higher than in the general population [19]. In addition, since ACL injury can lead to post-traumatic knee osteoarthritis at a relatively young age [2, 10, 20, 21], it can be an economic and physical burden to society [8]. It is especially significant in the military, since ACL injury can lead to termination of military service and premature discharge. The loss of military strength and combat power after such an injury is impossible to ignore. Therefore, to solve the problems caused by ACL injury, the most fundamental and effective strategy would be to prevent ACL injuries from occurring, as other sports groups have done [8, 22, 23].

This study showed that ACL injuries occurred more frequently through non-contact injury (84.5%) than through contact injury (15.5%), which is similar to the results of previous studies conducted in the sports setting, which reported non-contact injury as the cause of up to 70% of all ACL injuries [2, 8, 10, 12–17]. Since the activity that posed the highest risk of injury was soccer (54.1%) rather than military training (23.8%), the principle mechanism of injury may be more associated with competitive sports rather than military-specific activities. According to previous studies in sports settings, the incidence of ACL injury can be reduced to 50–80% when preventive programs are implemented [4, 8–11]. Thus, it could be assumed that implementing similar injury prevention programs in the military environment would reduce the incidence of ACL injuries.

The highest incidence of injury in this study was between 30 and 60 min after the start of exercise. Fatigue can increase anterior translation and increase angular error after running [9]. Fatigue caused by repetitive loading may contribute to the increased risk of non-contact ACL injury [24–28]. Because the soldiers strongly participate in exercise including competitive sports, just like combat, there would be considerable fatigue by 30 min after the start of exercise. We can presume that an injury time point between 30 and 60 min after the start of exercise is the most frequent time of injury although this was not statistically significant in our study. There were significant differences in age, weight, and BMI between the two groups in our study. High BMI and old age were the risk factors for lower extremity injuries in recruits [29–31]. The officer group tended to be injured earlier during exercise than the soldier group, likely due to having inferior physical strength and being older and heavier, resulting in less agility. Therefore, considering the degree of fatigue onset, the duration of exercise should be kept within 30 min (especially in competitive sports) and the officers should be more cautious about ACL injury.

There were several limitations of this study. First of all, it was a retrospective study based on medical records. Because some patients could have lapses in memory and not exactly recall the moment of injury, some errors may have been involved. Moreover, there was no control group available for comparison. The group characteristics could have been more accurately studied if a control group had been available for comparison. Furthermore, there were numerous environmental factors in the military setting that were difficult to control such as ground surface, equipment worn, etc., and it was impossible to reflect every variable in the final analysis.

## Conclusions

The main mechanism of ACL injury in the military environment was similar to that in the sporting environment, and most injuries occurred during exercise, rather than during military training. Soccer was most common activity at the time of the injury. Clinically, in the military environment, preventive strategies that minimize these risk factors could effectively reduce the incidence of ACL injuries.

This study could be a foundational study for the development of an ACL injury prevention program in the military environment, which could decrease the ACL injury rate significantly.

## Data Availability

Available.

## Abbreviations

ACL: Anterior cruciate ligament
BMI: Body mass index
MRI: Magnetic resonance imaging
